# The bending technique of a scoring balloon Aperta NSE leading to successful crossing into stents

**DOI:** 10.1002/ccr3.8524

**Published:** 2024-02-08

**Authors:** Naohiro Funayama, Keigo Kayanuma, Daisuke Sunaga, Makoto Furugen

**Affiliations:** ^1^ Department of Cardiology Hokkaido Cardiovascular Hospital Sapporo Japan

**Keywords:** percutaneous coronary intervention, scoring balloon

## Abstract

A new scoring balloon Aperta NSE has 3 longitudinal nylon elements mounted on the non‐compliant balloon surface. Although a high‐pressure balloon is usually used as a post‐dilation balloon in an implanted stent, it is difficult to pass into the stents because a balloon gets caught in stents in some cases. Aperta NSE has some grooves at elements; therefore, this balloon is bendable and shaped in an arc. The bent scoring balloon could eliminate interference between the balloon and the stents or lesions. Moreover, the point where the tip of the balloon contacts could change. As a result, it helps to improve crossability of this balloon. The bending technique of a scoring balloon Aperta NSE could lead to successful crossing into stents or complex lesions.

## 1 INTRODUCTION

Recently some scoring balloons are often used for complex lesions as a predilation balloon in percutaneous coronary intervention. Among scoring balloons, a new scoring balloon Aperta NSE has three longitudinal nylon elements mounted on the noncompliant balloon surface. Although this balloon was initially developed based on the concept of preventing a balloon from slipping, the scoring effect of the elements is currently expected. On the other hand, the deliverability of scoring balloons is lower compared with a conventional balloon.

Under expansion of implanted stents is well‐known to increase the risk of stent restenosis and stent thrombosis.^1^, ^2^ Although a high‐pressure balloon is usually used as a postdilation balloon in an implanted stent, it is difficult to pass into the stents because a balloon gets caught in stents in some cases. In this case, the bending technique of a scoring balloon Aperta NSE leading to successful crossing into a stent is described.

## 2 CASE PRESENTATION AND METHODS

**FIGURE 1:**
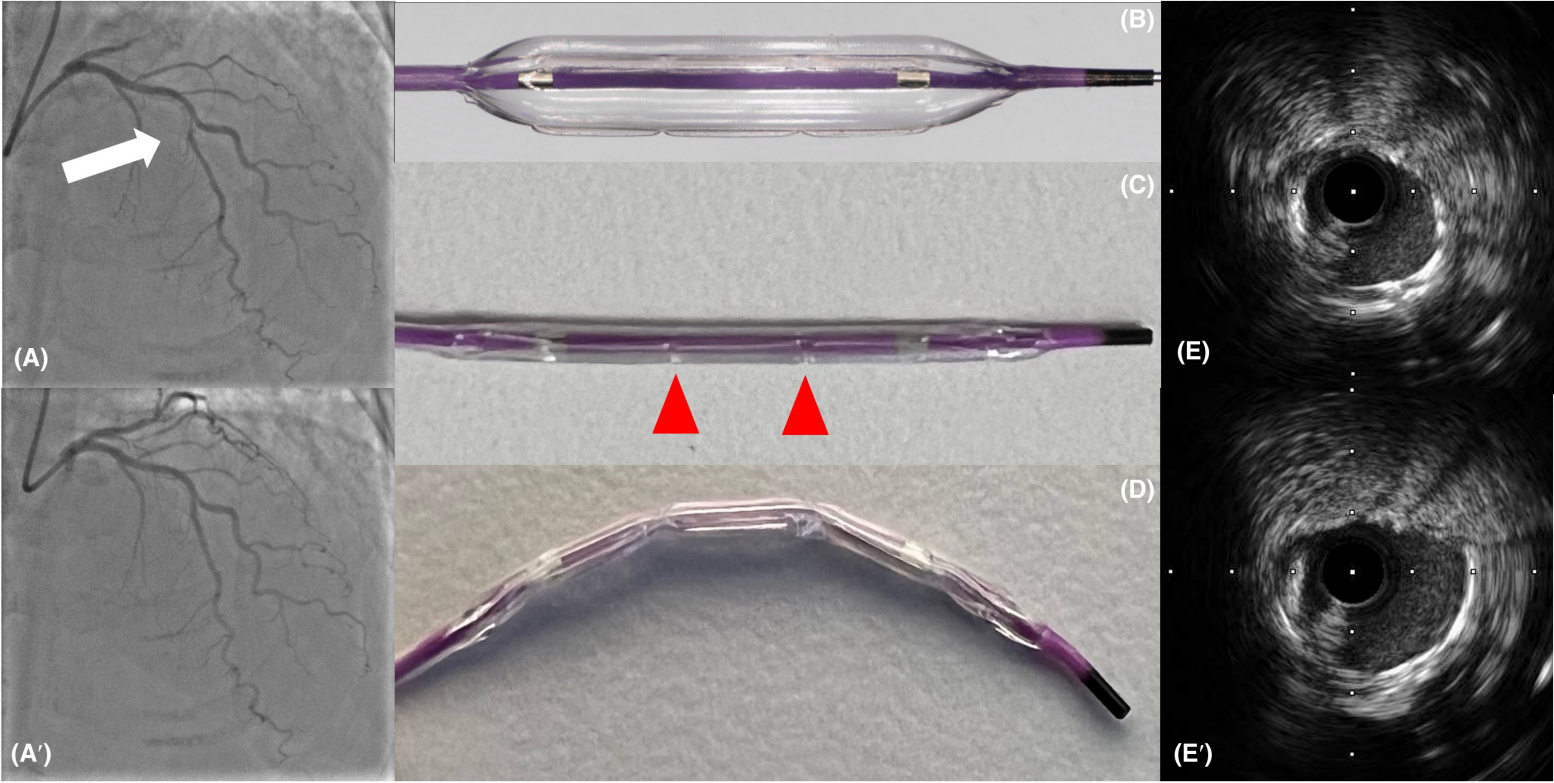
Images of the bent Aperta NSE. (A) Coronary angiography revealed severe luminal stenosis of the left anterior descending artery (white arrow). (A') Final coronary angiography showed good expansion of the stent. (B) Aperta NSE has the elements mounted on the balloon surface. (C) Aperta NSE has some grooves at elements (Red arrowhead). D: The bent and shaped balloon. (E) IVUS image poststenting. (E') IVUS image postdilatation.

A 72‐year‐old man was admitted to our hospital for the evaluation of effort‐induced chest pain. A 12‐lead electrocardiogram at rest demonstrated a normal sinus rhythm and no significant ST segment change. The coronary angiography revealed severe luminal stenosis of the left anterior descending artery (Figure 1A). We decided to perform elective percutaneous coronary intervention (PCI) for the middle LAD. We started PCI with a 6Fr backup type guide catheter (BESPA SS35, NIPRO, Japan) and 0.014‐inch guidewire. The lesion was predilated with an Aperta NSE scoring balloon (2.5/13 mm, NIPRO). An everolimus‐eluting stent (2.5/13 mm) was implanted at the target lesion. Intravascular ultrasound imaging showed the under expansion of the stent (Figure 1E). Although we tried to deliver the Aperta NSE into the stent to perform postdilatation, the balloon could not pass into the stent because of snagging (Video S1). We bent the Aperta NSE and shaped it in an arc (Figure 1D). After trying to deliver the balloon again, the bent Aperta NSE was able to pass into the stent (Video S2). Finally, we performed the postdilation with the balloon, and coronary angiography and IVUS showed good expansion of the stent (Figures 1A', E'). He was discharged in a good condition and his clinical outcomes were excellent at 6 months after intervention.

## 3 DISCUSSION

When we try to deliver a balloon into deployed stents, severe tortuosity and/or calcified lesions, a balloon may often get caught in stents or lesions. In particular, the deliverability of a once inflated balloon is often down and it is difficult to pass again. There are some solutions as the buddy wire technique,^3^ the usage of a Wiggle wire,^4^ a new postdilated balloon and a guide extension catheter. The buddy wire technique is effective, but there is the potential risk that the additional wire could pass through the malapposed stent struts. The Wiggle guidewire has a unique construction with a series of corrugations or bends near the distal end and helps some devices pass into stents or tortuous vessels. Although the usage of a new postdilated and guide extension catheter might be an alternative technique, it is difficult to deliver in stents in case the tip of the balloon contacts the stent struts. In all technique, extra costs and time are required. The Aperta NSE has three longitudinal nylon elements mounted on the balloon surface and is used as a predilatation scoring balloon (Figure 1B). In addition, this balloon is a noncompliant balloon; therefore, it could be used as a postdilatation balloon. Aperta NSE has some grooves at elements (Figure 1C); therefore, this balloon is bendable and shaped in an arc (Figure 1D). The bent scoring balloon could eliminate interference between the balloon and the stents or lesions. Moreover, the point where the tip of the balloon contacts could change. As a result, it helps to improve crossability of this balloon in some cases. This unique technique is very simple to perform without extra costs and time.

## 4 CONCLUSION

The bending technique of a scoring balloon Aperta NSE could lead to successful crossing into stents or complex lesions.

## AUTHOR CONTRIBUTIONS

**Naohiro Funayama:** Conceptualization; data curation; investigation; project administration; visualization; writing – original draft; writing – review and editing. **Keigo Kayanuma:** Writing – review and editing. **Daisuke Sunaga:** Writing – review and editing. **Makoto Furugen:** Writing – review and editing.

## FUNDING INFORMATION

The author's received no funding for the research, authorship and publication of this article.

## CONFLICT OF INTEREST STATEMENT

None.

## ETHICS STATEMENT

The article describes a case report and no additional permission from our Ethics Committee was required.

## CONSENT

Written informed consent was obtained from the patient to publish this report in accordance with the journal's patient consent policy.

## Supporting information


**Video S1:** The Aperta NSE was not able to pass into the stent.

**Video S2:** The bent Aperta NSE was able to pass into the stent.

## Data Availability

All data are available in this published article.
